# Editorial: Action mechanisms of traditional medicinal plants used to control type 2 diabetes or conditions of metabolic syndrome, volume II

**DOI:** 10.3389/fphar.2023.1267677

**Published:** 2023-09-12

**Authors:** Adolfo Andrade-Cetto, Md. Shahidul Islam, Michael Heinrich, Vincenzo De Feo

**Affiliations:** ^1^ Department of Cellular Biology, School of Sciences, National Autonomous University of Mexico, Mexico City, Mexico; ^2^ Department of Biochemistry, School of Life Sciences, University of KwaZulu-Natal, Westville Campus, Durban, South Africa; ^3^ School of Pharmacy, Faculty of Life Sciences, University College London, London, United Kingdom; ^4^ Department of Pharmacy, University of Salerno, Fisciano, Italy

**Keywords:** metabolic syndrome, type 2 diabetes, medicinal plants, traditional medicine, diabetes

Metabolic diseases (MD) are a burden for modern societies, they affect individuals’ health as well as public health and the country’s economies. The WHO estimates that most of the world’s population lives in countries where overweight and obesity kills more people than underweight. At the same time, diabetes mellitus is now among the top ten global causes of death (WHO, 2023). MD are a cluster of conditions (increased blood pressure, high blood sugar, excess body fat around the waist, and abnormal cholesterol or triglyceride levels) that when they occur together are called; Metabolic syndrome and type 2 diabetes; mainly defined by insulin resistance (IR) in the presence of abnormal insulin secretion.

Traditional medicines worldwide are facing a challenge to treat MD that has existed clinically for a long time. However, with the changes in lifestyle (lack of exercise, poor nutrition) and simultaneously increase in life expectancy, these conditions are growing in epidemic proportions and threatening the lives of millions of people. While the life expectancy has increased at an impressive scale, the increase in the average years of a healthy life lags behind. Although many of the traditional systems have recognized these problems for a long time within specific cultural interpretations of disease states, currently chronic metabolic diseases play an important role in these systems, since healers and primary care providers are an important source of healthcare, also for metabolic syndrome and related conditions. At the same time, advances in sciences allow a better understanding and labeling of the diseases that had been previously incorporated in the traditional systems.

The goal of this Research Topic was to contribute to understanding how medicinal plants are facing the challenge to treat MD and DM in our “modern” societies. In this aspect seven articles are included in the Research Topic, here with we introduce some of them: Jia et al., demonstrate the effects of Alismatis Rhizoma [*Alisma plantago-aquatica* subsp. *orientale* (Sam.) Sam.] on metabolic syndrome with a focus on bioactive triterpenoids using a metabolomic and lipidomic approach. They conclude that Alismatis rhizome, a source of traditional Chinese medicine, can treat metabolic syndrome mainly by inhibiting energy metabolism, amino acid metabolism, and regulating bile acid to reduce phospholipid content. Li et al. reported the hypoglycemic effects as well as underlying mechanisms of actions of Chinese polyherbal formula JinQi Jiangtang in the form of oral tablet. From the results of this study, it has been shown that Palmatine, a key active metabolite of JinQi Jiangtang, significantly stimulated the phosphorylation of fibroblast growth factor receptor 1 (FGFR1) and upregulated the glucose transporter 1 (GLUT-1) expression to increase glucose uptake in insulin resistant HepG2 cells and reduced hyperglycemia in high-fat diet-fed streptozotocin-induced diabetic mice. The pharmacological findings of this study have been investigated further using molecular docking, qPCR and Western blotting techniques.

The effects of quercetin (a widely distributed flavonoid in the plant kingdom) over Polycystic Ovary Syndrome (PCOS), was reviewed by Ma et al. PCOS is a multifactorial endocrine disease, the main clinical features are hyperandrogenemia, ovarian enlargement, no ovulation or oligomenorrhea, accompanied by endocrine abnormalities, metabolic abnormalities, and reproductive dysfunction, the review concludes that quercetin *in vitro* can relieve oxidative stress and apoptosis of ovarian cells, it also can effectively reduce serum testosterone, luteinizing hormone (LH), the LH/FSH (follicle stimulating hormone) ratio, fasting blood glucose and fasting insulin. The authors recommend, for the future, the need for more clinical trials applying the flavonoid in PCOS patients.

Ye et al. demonstrate that Jiangtang Sanhao formula (JTSHF), a prescription for treating patients with diabetes mellitus (DM) in traditional Chinese medicine may ameliorate skeletal muscle IR in diabetes mice. They claim that its underlying mechanism could be attributed to stimulating the expression and translocation of GLUT4 by activating the AMPKα/SIRT1/PGC-1α signaling pathways.

Ethnopharmacology in the 21st century faces major challenges solving the needs for improved treatments of Metabolic Syndrome and Type 2 diabetes, we invite you to read the seven works, which will certainly help in better understanding how, traditional systems are dealing with these diseases.
